# Vision transformer network discovers the prognostic value of pancreatic cancer pathology sections via interpretable risk scores

**DOI:** 10.1007/s12672-025-03547-3

**Published:** 2025-09-03

**Authors:** Zhiyong Peng, Yue Zhang, Tianchi Zhou, Wenjie Shi, Ya Wang, Maciej Pech, Georg Rose, Maximilian Dölling, Katrin Hippe, Roland S. Croner, Yi Zhu, Ulf D. Kahlert

**Affiliations:** 1 School of Optoelectronic Engineering, Guilin University of Electronic Technology, Guilin, China; 2 Molecular and Experimental Surgery, Clinic for General-, Visceral-, Vascular- and Transplantation Surgery, Medical Faculty and University Hospital Magdeburg, Otto-von-Guericke University, Magdeburg, Germany; 3 Clinic for Radiology and Nuclear Medicine, University Hospital Magdeburg, Magdeburg, Germany; 4 Research Campus Stimulate, Otto von Guericke University, Magdeburg, Germany; 5 Institute of Pathology, Medical Faculty and University Hospital Magdeburg, Otto-von-Guericke University, Magdeburg, Germany; 6 Department of Gastroenterological Surgery, The Affiliated Hospital of Jiaxing University, Jiaxing, Zhejiang China

**Keywords:** Vision transformer, Pancreatic cancer, Machine learning, Risk score, Personalized medicine

## Abstract

Pathological sections hold rich diagnostic information, yet their prognostic potential is underutilized. This study leverages deep learning to predict outcomes, advancing precision oncology of pathological sections with focus on pancreatic cancer. We analyzed H&E-stained whole section images of 125 cases from public databases as well as 28 real-world patients with pancreatic cancer and precancerous lesions. After image preprocessing, we identified and selected representative patches for subsequent analysis. We develop a modified visual transformer (ViT) model with spatial attention and fine-tuned on ImageNet2012, which was subsequently used to predict the survival times of the corresponding patients and to calculate risk scores. The modified ViT model demonstrated strong predictive accuracy for patient prognosis, with C-indices of 0.79 and 0.82 for Overall Survival (OS) and Disease Free Survival (DFS) in the test set and 0.62 in the validation set. Risk scores correlated well with patient survival, showing clustering between 0.17 and 0.95, aligning with a median survival of 24 months. Higher risk scores were associated with worse clinical prognosis, including shorter survival times and increased tumor recurrence risk, validated across all datasets. The model’s AUCs for OS and DFS prediction reached 0.847/0.849 in the training set and 0.813/0.834 in the test set, confirming its high accuracy and potential for clinical application in risk stratification and prognosis prediction. ViT network can discover the prognostic value of pancreatic cancer pathology sections via interpretable risk scores, providing a new insight for prognosis evaluation as well as opens new technology building on existing clinical diagnostics.

## Introduction

Pancreatic cancer (PC), primarily pancreatic ductal adenocarcinoma (PDAC), has seen a significant global increase in both incidence and mortality over the past 25 years, making it the third-leading cause of cancer-related deaths in the United States [1]. With increasing quqntitiy in diagnosis rates, it is anticipated that PDAC will ascend to the position of the second most common cause of cancer deaths in Western nations in the forthcoming decades [2]. Unlike many other cancers, PDAC presents with extremely poor outcomes, as about 50% of new cases are diagnosed in the metastatic stage with an average survival of less than a year, and overall five-year survival rates hover around a mere 11% [1, 3]. This dismal prognosis is primarily due to the non-specific symptoms that frequently result in late diagnosis, while early-stage PDAC patients can experience dramatically higher survival rates, such as approximately 80% for those with stage I disease [4], emphasizing the necessity of risk stratification and individualized precision treatment to address tumor heterogeneity, even among patients within the same histopathological classification stage.

Recent advancements in artificial intelligence (AI) have significantly impacted medical fields, extending to genomics [5], radiomics [6], and digital pathology (DP) [6], transforming diagnostic processes and enhancing personalized medicine strategies [7]. DP involves digitizing histopathology slides using whole-slide scanners and analyzing these images with computational methods, which was first introduced around the 1990s [8], the introduction of AI technologies such as machine learning (ML) and deep learning (DL) can significantly enhance the precision and speed of diagnosing diseases by automating the analysis of complex medical images and identifying subtle patterns. It also contributes to prognostic evaluations that predict disease progression and outcomes, thereby supporting personalized treatment planning [9, 10]. ML involves algorithms that learn from data and make predictions, while DL is a subset of ML that uses layered neural networks to analyze various levels of data abstraction, offering the advantage of handling large, complex datasets more effectively and achieving greater accuracy in tasks such as image and speech recognition [8, 11]. Based on these computational algorithms, increasing research is applying DL in DP to stratify tumor risk and predict prognosis. I.e.a previous study developed a DL-based tool for analyzing cellular spatial organization in lung adenocarcinoma pathology images, revealing that specific cell distribution patterns within the tumor microenvironment (TME) are predictive of patient survival and correlate with the activation of key biological pathways [12]. Shi et al. [13]. also presented an DL framework that effectively decodes hepatocellular carcinoma pathology images for risk stratification and prognosis prediction, demonstrating superior predictive power over traditional clinical staging and revealing key phenotypic and genomic correlations with patient outcomes.

In this research, we leverage deep learning (DL) algorithms to analyze pathological whole-slide images (WSIs) of pancreatic cancer (PC), aiming to develop a novel prognostic model that predicts disease outcomes with high accuracy. What sets this work apart is our focus on integrating high-resolution WSI data with advanced DL techniques to identify subtle and complex tumor features that may not be detectable through conventional methods. Unlike previous models that may rely on a limited subset of clinical data, our approach emphasizes a comprehensive, image-based analysis, which can be used to predict patient outcomes and guide risk-stratified treatment plans. By incorporating individual tumor characteristics and patient profiles, this model aims to provide a more personalized and precise approach to diagnosis, advancing current research by enabling more effective, data-driven interventions for pancreatic cancer.

## Materials and methods

### Patient cohort enrollment

**Fig. 1:**
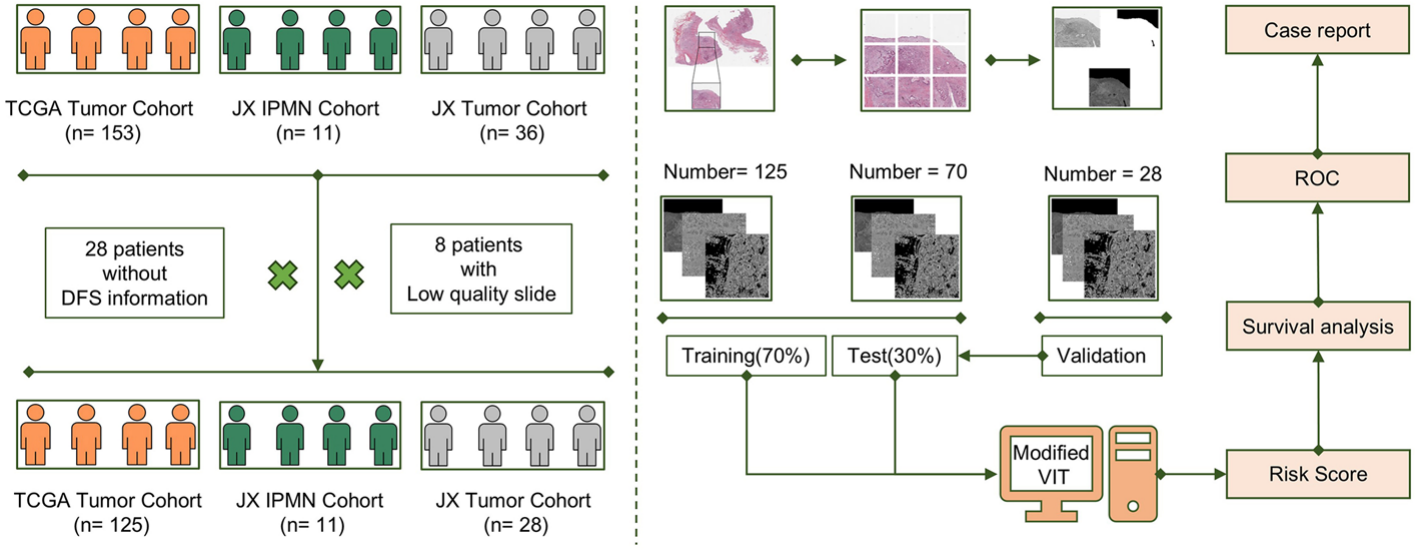
Workflow of study

This study included data on patients with pancreatic cancer and precancerous lesions from The Cancer Genome Atlas (TCGA, https://portal.gdc.cancer.gov) and Jiaxing College Hospital. We firstly, obtained malignant pathology sections and follow-up information of 153 pancreatic cancer patients from TCGA, including Overall Survival (OS) and Disease-Free Survival (DFS). Due to missing DFS information in 27 patients, these patients were excluded, resulting in a residual inclusion of 125 patients. In addition, we included pathologic sections from 11 patients with precancerous lesions as a standard reference cohort, and assumed that these patients had a survival period of 50 years. Secondly, we also collected malignant pathology sections from 36 pancreatic cancer patients from Jiaxing College Hospital, and after screening, we removed 8 samples with blurred images or staining contamination, and retained 28 patients’ pathology sections, the baseline information was shown in Table 1.The work flow shown in Fig. 1.

**Table 1:** Baseline information of enrolled patients

Variable	Group		*p* value
	Male	Female	
Age (Years)	65.07 ± 8.21	69.57 ± 9.43	0.029
Tumor size (mm)	31.00 ± 13.20	37.07 ± 9.70	0.117
Positive Nodes	1.07 ± 2.16	1.93 ± 2.73	0.547
CEA	2.74 ± 1.80	4.07 ± 5.63	0.747
Perineural Invasion			0.418
No	6	3	
Yes	8	11	
Chronic Pancreatitis			0.589
No	3	1	
Yes	11	13	
Grade			0.250
I	0	2	
II	8	5	
III	6	7	

### Histopathologic samples and representative images

All pathological sections of the enrolled patients were hematoxylin and eosin (H&E)-stained whole slide images (WSIs). To ensure that each cropped block contained complete pathological information, each WSI was cropped into 20,000 × 20,000-pixel image blocks. Subsequently, the most representative block from each sample was reviewed and selected by two certified pathologists as the representative image of that patient for downstream analysis. In total, 125 representative image blocks of pancreatic cancer were extracted from the TCGA pancreatic cancer cohort, while 70 blocks of precancerous lesions and 28 blocks of pancreatic cancer were collected from the standardized cohort and the pancreatic cancer cohort of Jiaxing College, respectively.

For model construction, we employed a convolutional neural network (CNN) based on the ResNet50 architecture, initialized with ImageNet-pretrained weights and fine-tuned on the histopathology datasets. All representative image blocks were resized to 512 × 512 pixels and normalized before being fed into the network. The model was trained using the Adam optimizer (initial learning rate 1 × 10^-4, batch size 32) with an early stopping strategy to prevent overfitting. Data augmentation techniques, including random rotation, flipping, and color jitter, were applied during training. The final fully connected layer was modified to output binary predictions (precancerous vs. cancerous) with a softmax activation function. Model performance was evaluated on the independent Jiaxing College cohort using accuracy, area under the receiver operating characteristic curve (AUC), sensitivity, and specificity as metrics.

### Modified ViT modeling output risk score

**Fig. 2:**
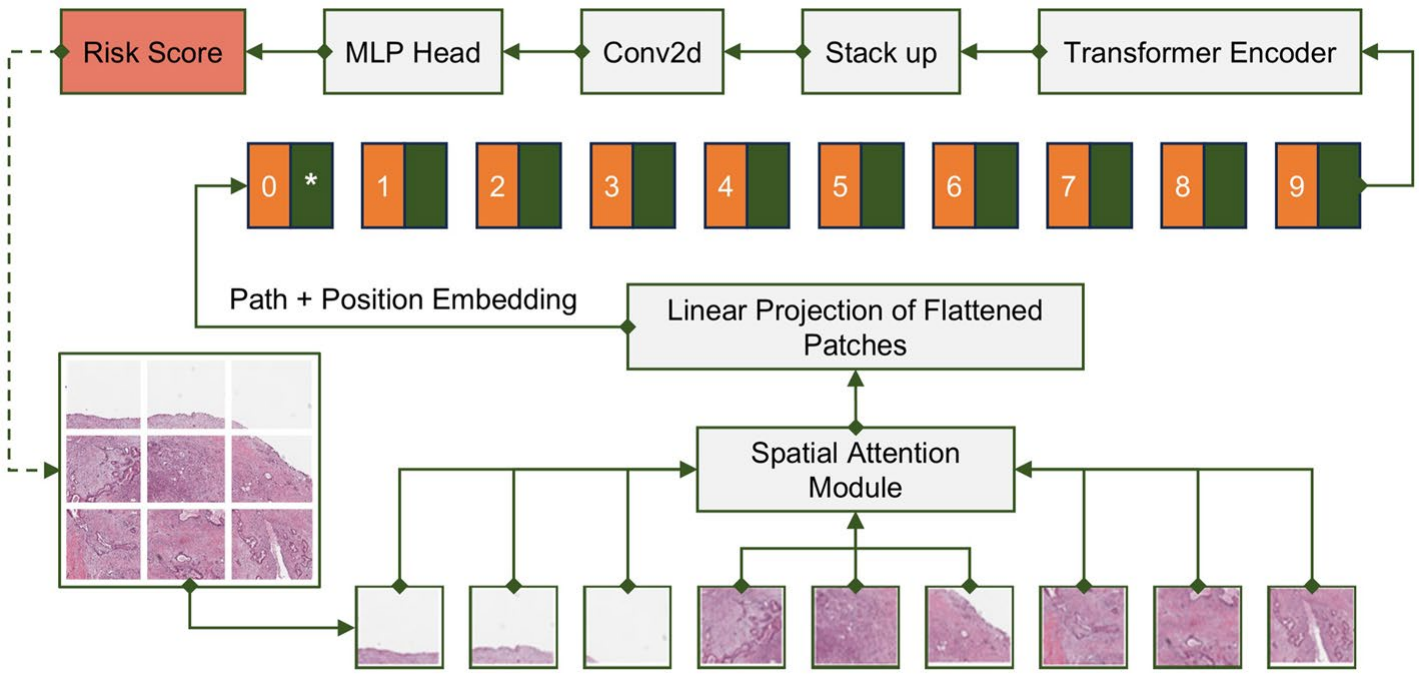
The framework of Modified Vit model

We defined the outcome events as OS and DFS, and in order to predict the survival probability of pancreatic cancer patients, we trained an improved deep learning model based on the Vision Transformer (ViT) algorithm on the pathology slices. We added a spatial attention mechanism at the data input of the improved ViT model and stacked the attention weights of the 12 outputs of the Encoder module to improve the capability of the network to select features from pathology images (Fig. 2). The training and test sets of the model included a total of 195 sections, consisting of 125 malignant pathology sections from TCGA pancreatic cancer patients and 70 precancerous tissue sections, which were randomly assigned in a 7:3 ratio. To mitigate the uniform staining problem, all images were converted to grayscale, and a mask was applied to extract the tissue regions. Based on this, the improved ViT model was fine-tuned. Model pre-training was performed on the ImageNet2012 dataset. The model enables us to compute a prognostic-related risk score for each image block. To evaluate the model’s performance more robustly, we employed 5-fold cross-validation and reported the mean of the consistency index (C-index) across multiple runs. The C-index was used as the primary metric to assess predictive efficacy. This revised approach improves the reliability and generalizability of the model’s performance.

**Fig. 3:**
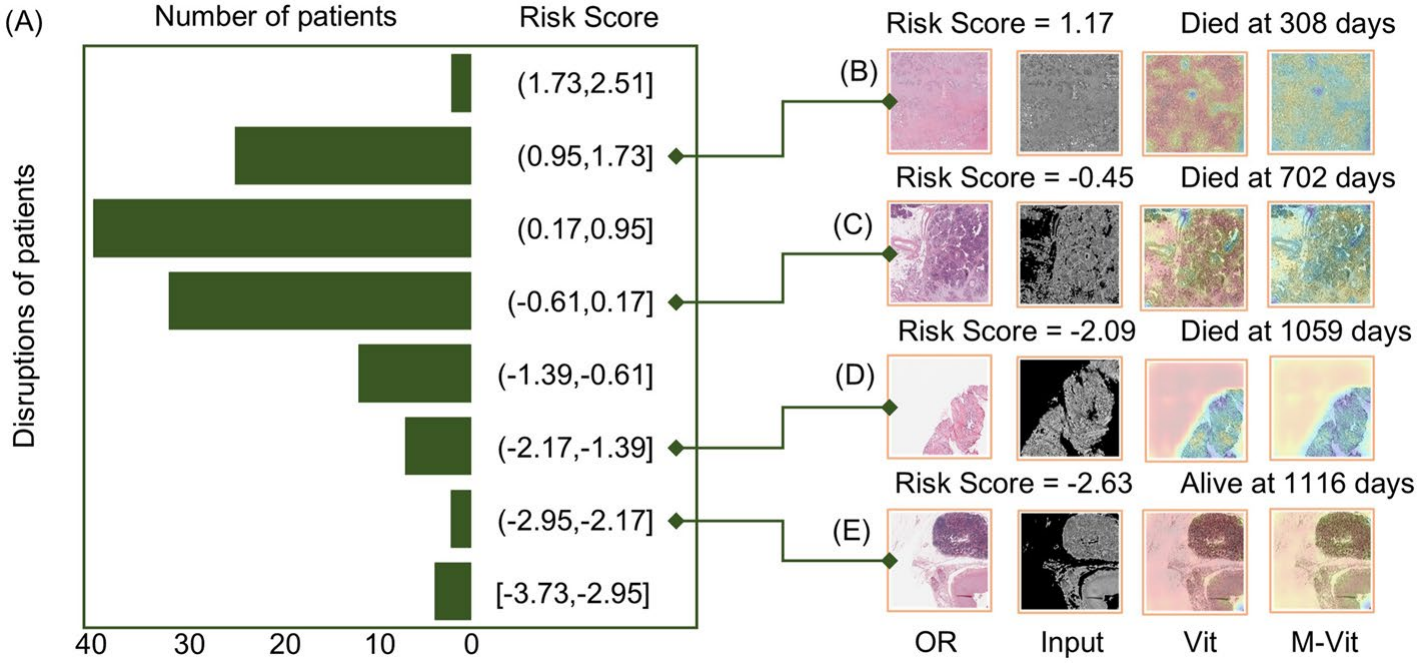
Model-associated riskscore distribution and case visualization. **A** Risk Score and Patient Count Distribution. **B** case died at 308 days with 1.17 score. **C** case died at 702 days with − 0.45 score. **D** case died at 1059 days with − 2.09 score. **E** case died at 1116 days with − 2.63 score

**Fig. 4:**
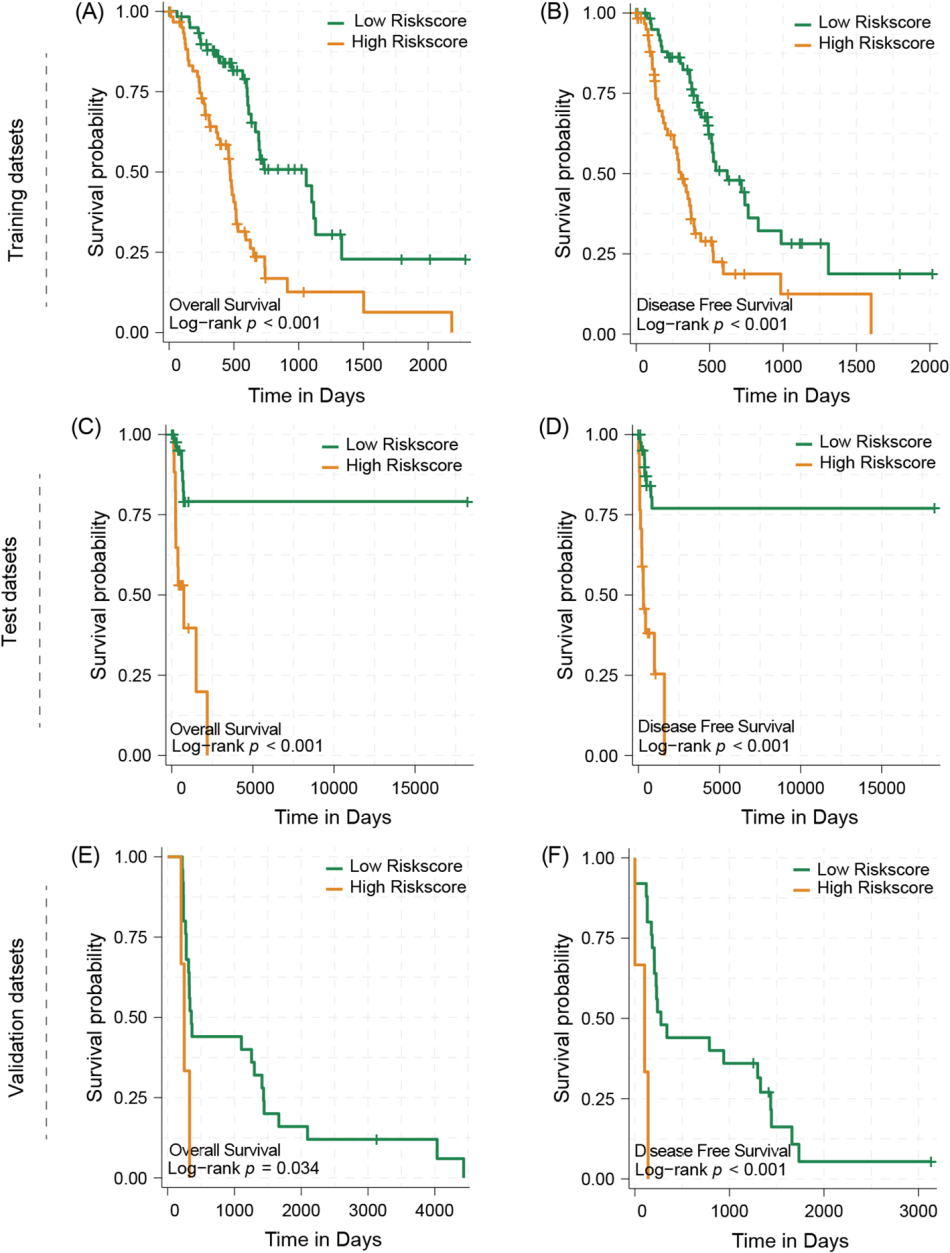
The survival analysis of different risks core group in training datasets, test datasets and validation datasets. **A** OS difference for Low-and High Risk in the training dataset. **B** DFS difference for Low-and High Risk in the training dataset. **C** OS difference for Low-and High Risk in the test dataset. **D** DFS difference for Low-and High Risk in the test dataset. **E** OS difference for Low-and High Risk in the validation dataset. **F** DFS difference for Low-and High Risk in the validation dataset

**Fig. 5:**
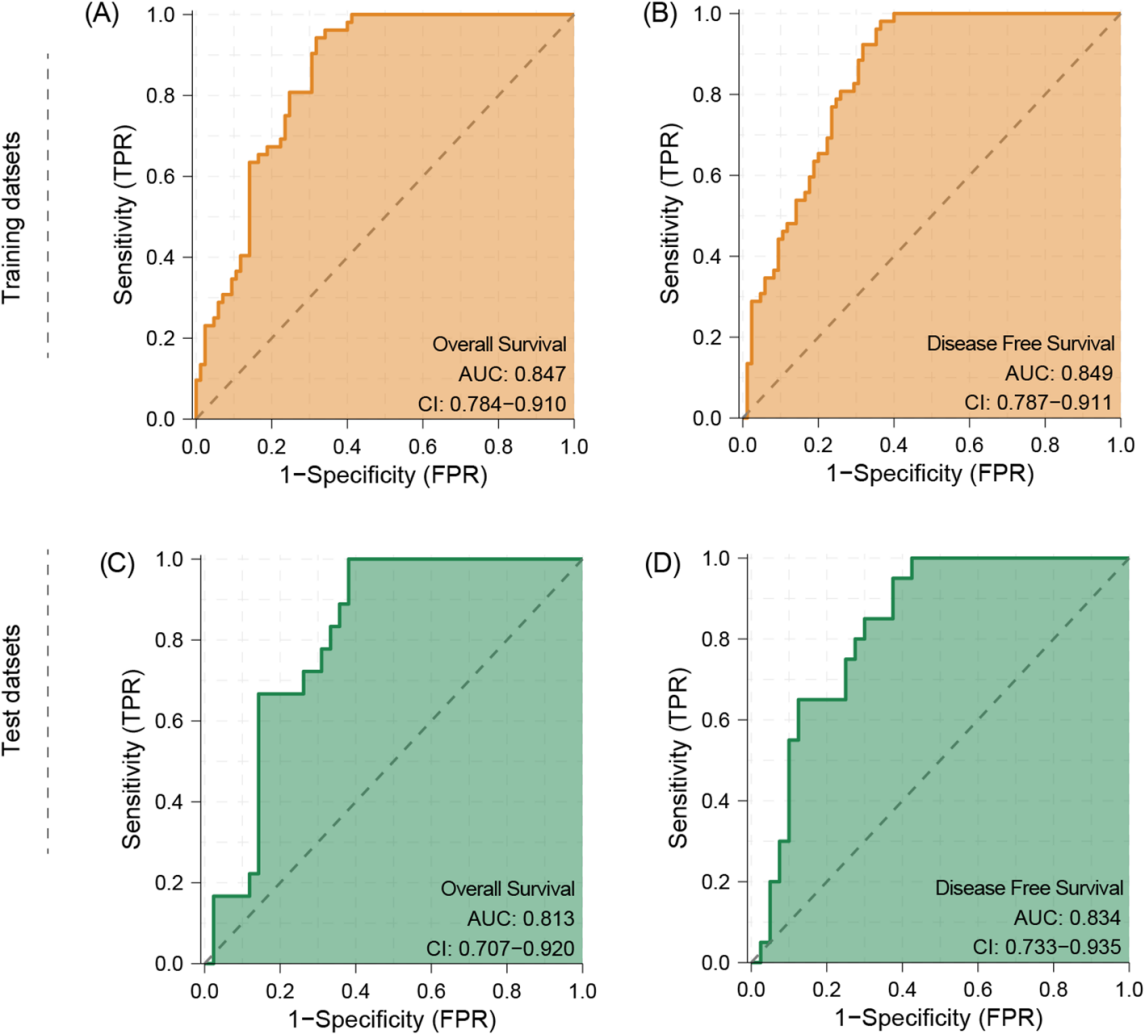
ROC for OS and DFS prediction in training and test datasets. **A** ROC for OS in the training dataset. **B** ROC for DFS in the training dataset. **C** ROC for OS in the test dataset. **D** ROC for DFS in the test dataset

### Riskscore and prognosis

To evaluate the prognostic value of the risk score, we similarly included OS and DFS as endpoint events. Risk scores output from the model were grouped according to the median value, and patients above the median value were defined as the high-risk group, while others were included in the low-risk group. log-rank test was used to analyze the difference in survival between groups, and area under the ROC curve was used to assess the accuracy of the risk scores for prognostic evaluation.

## Results

### Modified ViT model can effectively predict clinical prognosis of patients

The consistency index is an important index to assess the performance of the prediction model, and the higher the value, the better the agreement between the survival time ranking predicted by the model and the actual survival time. By training and testing the model, the modified ViT model performed well in assessing the risk of OS and DFS of pancreatic cancer patients. In the test set, the average C-index of the model reached 0.79 and 0.82, respectively, indicating its high predictive accuracy. In the independent validation set, the C-index of the model are 0.62 and 0.64 respectively, which is still valuable for application in risk stratification analysis, despite the decrease in accuracy (Table 2).

**Table 2:** 5-Fold Cross-Validation C-index for OS and DFS in the test validation sets

Endpoints	Epoch 2	Epoch 3	Epoch 4	Epoch 5	Epoch 6	Average
OS C-index	0.78	0.78	0.77	0.80	0.82	0.79
DFS C-index	0.80	0.82	0.84	0.84	0.86	0.82

### Model-associated riskscore distribution and case visualization

To further explore the relationship between model output risk scores and patient prognosis, we statistically analyzed the distribution of risk scores of patients in the TCGA cohort. The results showed that the majority of patients had scores clustered between 0.17 and 0.95, which corresponded to a survival duration of approximately 24 months, a result consistent with the median survival time of pancreatic cancer patients. In addition, we visualized representative patient sections from different risk score intervals. The prognosis of patients gradually improved as the risk score decreased (Fig. 3). Notably, the clarity of the section images showed a negative correlation with the patient risk score, i.e., tissue sections from low-risk patients tended to have more defined pathological features, which may be related to the differences in the tumor microenvironment and pathological status. This phenomenon suggests that the risk score of the model not only reflects the survival duration, but also may be potentially associated with histologic features, providing a new direction for further research.

### High risk riskscore leads to poor prognosis

To validate the clinical application value of riskscore in prognosis prediction, we stratified the survival data of patients in the training set, test set and validation set. The results showed that compared with low-risk patients, high-risk patients were usually accompanied by a severely worse prognosis, both in terms of OS and DFS(*p* < 0.05). Specifically, high-risk patients usually exhibited shorter survival time and higher risk of recurrence (Fig. 4). This trend was validated in all datasets, indicating that the riskscore output from the modified ViT model has good stability and generalizability across different datasets.

### Riskscore predicts patient prognosis

To further evaluate the predictive efficacy of riskscore on prognostic evaluation of pancreatic cancer patients, we quantified its accuracy by subject operating characteristic curve (ROC) and area under the curve (AUC). In the training set, the AUCs of risk scores for OS and DFS prediction were 0.847 and 0.849, respectively; in the test set, the AUCs were 0.813 and 0.834, respectively (Fig. 5). These results suggest that the risk scores output from the modified ViT model have high accuracy in distinguishing between high- and low-risk patients. However, in the validation set, ROC curves could not be plotted due to the small number of patient events.

## Discussion

Through our research, we have employed an enhanced DL model based on the ViT algorithm to accurately differentiate between PC and non-cancerous lesions in pathology slices. Building on this, we developed a risk model whose risk score demonstrates strong predictive capabilities. Furthermore, tissue sections from low-risk PC patients exhibited more defined pathological features. This observation suggests that the model’s risk score not only predicts survival duration but may also be linked to specific histological characteristics, offering a promising avenue for further research.

The ViT adapts the transformer architecture originally for natural language to process visual data by segmenting images into patches treated as tokens. This allows ViT to globally analyze relationships across the image, providing a major advantage in handling complex patterns more effectively than traditional convolutional neural networks (CNNs) [14, 15]. Zhai and his colleagues extended the application of ViT for image recognition by focusing on scaling strategies that enhance performance, effectively showing how ViTs can outperform traditional CNN architectures in larger-scale and more complex image classification tasks [15]. Currently, ViT is widely applied in digital pathology for diagnostic classification and prognosis prediction, demonstrating its utility in enhancing detailed image analysis within the medical field. A previous study utilizes DL, particularly ViT to automate the detection and accurate Gleason grading of prostatic adenocarcinoma from WSIs, enhancing pathologists’ ability to diagnose and treat prostate cancer effectively [16]. Another recent study introduced a customized ViT algorithm, which can effectively stage HER2 expression in breast cancer using H&E stained images, outperforming conventional models and significantly reducing the need for expensive and complex immunohistochemical staining, thus enhancing diagnostic accessibility in low-resource settings [17].On the other hand, while MIL (Multiple Instance Learning) remains a widely used and resource-efficient method for WSI analysis, we opted for a block-based ViT (Vision Transformer) approach for several reasons. MIL models typically require feature aggregation from multiple image patches, which can increase computational cost, especially when handling large datasets. In contrast, the block-based ViT directly processes image blocks through self-attention, allowing for efficient capture of both local and global context, with less computational overhead during aggregation. Direct performance comparisons show that the ViT model outperforms MIL-based approaches, particularly in capturing long-range dependencies and tissue structure context, which are crucial for accurate prognostic predictions. The ViT’s superior ability to model intricate spatial relationships in high-resolution WSI data justifies its use over traditional MIL methods, especially when scalability and fine-tuning on diverse datasets are considered. Furthermore, our approach is validated through comprehensive comparisons with relevant state-of-the-art models, including those that use graph convolutional networks (GCN) for multi-modal integration, such as in breast cancer prognosis studies. Unlike GCN methods, which rely on feature aggregation across different data modalities, our ViT model integrates local and global tissue features within the image itself, offering a more efficient and scalable solution for high-resolution WSI analysis. This unique characteristic allows our model to achieve better performance in terms of both predictive accuracy and computational efficiency, making it a promising alternative for clinical applications in pancreatic cancer prognosis [18, 19].

Currently, the application of DL in PC research has expanded into the radiomics field [20, 21], yet studies in DP remain limited. In a previous study, an anatomy-guided transformer-based deep classification model was developed which achieves a sensitivity of 95.2% and specificity of 95.8%, significantly outperforming radiologists in detecting and classifying resectable pancreatic masses using non-contrast CT scans, offering a potential new tool for large-scale pancreatic cancer screening [20]. In another study, a DL model called PANDA successfully detects and classifies pancreatic lesions using non-contrast CT scans, achieving an AUC of 0.986–0.996, a sensitivity of 92.9%, and specificity of 99.9% across multiple validation scenarios, significantly surpassing radiologist performance [21]. In addition, Chen et al. [22]. also introduced a new DL algorithm with a channel and spatial self-attention (CS) module that enhances the diagnostic accuracy of PC pathology images by significantly outperforming existing methods and improving key metrics on specialized datasets. Recently, rapid on-site evaluation based on an altered shuffled instance-aware VIT approach of pathology images for PC was achieved, indicating in the great potential of ViT technologies to transform pathologist work burden. Improving the accuracy of preoperative diagnostics of tumor metastasis, especially tumor metastasis in lymph nodes is of highest clinical need as it may help to streamine surgical planning and individualize neoadjuvant chemotherapy regimes. ViT has recently been shown to allow tumor metastasis in lymph nodes of PC, further calling for prospective clinical studies using VIT-diagnostics. Indeed, a very recenty work on liver cancer showed the enormous potential of VIT model HEROVision to stratify patients suitable for surgical resection vs. minimal invasive therapy, both intended for curation of the patient. Furthermore, some scholars have effectively combined a self-supervised CNN feature extractor with a ViT to classify WSIs of PDAC by analyzing patches, achieving higher patch-level and WSI-level prediction accuracy compared to using a conventional ImageNet feature extractor [23]. However, these models do not provide prognosis predictions for PC patients, whereas our model accurately forecasts outcomes with C-indices of 0.62 in the independent validation set, aligning with the risk score distribution observed in individual case visualizations.

Interestingly, in our study, tissue sections from low-risk patients displayed more distinct pathological features, suggesting that the model’s risk score not only forecasts survival duration but may also correlate with specific histological traits, reflecting the spatial characteristics of the TME. With the advancement of DP, scholars recommend using DL to map ‘Tumor-Tumor Infiltrating Lymphocytes (TILs)’ in WSIs, which may further enhance our understanding of tumor-immune interactions across various cancers and aid in the prediction of clinical outcomes [24]. A previous study has developed CNN-based analysis pipelines that generate combined maps of cancer regions and tumor-TILs in breast cancer WSIs, providing detailed insights into their spatial relationships and structural patterns [25]. In addition, Saltz et al. [26]. utilized DL to computationally map TILs in 5,202 H&E-stained slides across 13 cancer types from TCGA, revealing that these TIL patterns correlate with pathologist assessments, molecular estimates, and patient outcomes. Moreover, in a recent study, Zhao et al. [27]. developed an ML model that accurately detects and classifies tertiary lymphoid structures (TLSs) in H&E stained slides from PDAC, showing that the presence of mature TLSs correlates with better survival outcomes, thereby highlighting the model’s potential as a predictive biomarker for PDAC prognosis. These studies demonstrate that analyzing WSIs using DL and ML models offers a viable method for assessing interactions between tumor cells and theTME, providing valuable insights for improving cancer diagnosis and prognosis predictions [12].

## Conclusion

In summary, we developed a ViT-based DL model that effectively distinguishes between PC and non-cancerous lesions, offering a tool for risk stratification and precision treatment of PC. However, our study has several limitations. First, we did not include clinical or genomic data, which could provide additional information on tumor heterogeneity. Second, due to the limited sample size, the effectiveness of our model decreased in the validation set, necessitating further training and validation with larger samples from more centers involving different ethnicity to ensure model stability. Lastly, while we observed that different risk scores correlate with distinct histological traits, we did not further identify the conditions of the TME in PC patients. This limitation underscores the necessity for additional research, which could potentially yield valuable insights into the prognosis and immunotherapy of PC through DL analysis of WSIs.

## Data Availability

The datasets analysed during the current study are available from the corresponding author on reasonable request.
